# The Invasive Plant, *Alliaria petiolata*, Is an Ecological Trap for the Native Butterfly, *Anthocharis midea*, in North America

**DOI:** 10.3390/insects16040331

**Published:** 2025-03-21

**Authors:** Danielle M. Thiemann, Don Cipollini

**Affiliations:** Department of Biological Sciences, Wright State University, Dayton, OH 45435, USA; thiemannd1@xavier.edu

**Keywords:** invasive plants, chemical defenses, oviposition, larval performance, Brassicaceae, abiotic stress

## Abstract

The introduction of novel plant species to new habitats may have consequences for native herbivores. We examined the impact of *Alliaria petiolata*, a Eurasian invader of forest understories and edges in North America, on adult oviposition behavior and on larval preference and the performance of *Anthocharis midea*, a univoltine butterfly that specializes on plants in the Brassicaceae. We compared the usage of *A. petiolata* by this butterfly to that of one of its common native hosts, *Cardamine concatenata*, and additionally explored the impact of drought and larval age on the larval success of these hosts. In the field, adults oviposited on the native host preferentially earlier in the season, but they strongly preferred *A. petiolata* later in the season and laid more eggs overall and in multiples more often on this plant. Larvae strongly preferred to feed on leaves of *C. concatenata* over *A. petiolata* in the laboratory and survived to pupation at a high rate on it. Conversely, larvae fed little and died when offered *A. petiolata*. Larvae preferentially chose tissues of droughted *C. concatenata* over well-watered plants, but drought stress had no impact on larval mass and survival to pupation. Larvae showed no preference for droughted or non-droughted *A. petiolata* and while drought stress tended to extend survival on this plant, all larvae still died on it. Older larvae were no better at feeding and surviving on *A. petiolata* than young larvae. Our results indicate that *A. petiolata* currently serves as an ecological trap for *A. midea*, being both highly attractive to ovipositing adults yet lethal to larvae. This effect could cause both local declines in the abundance of this butterfly and drive selection for the altered behavior of adults and larvae to either avoid or better tolerate this plant.

## Simple Summary

Invasive plant species can have numerous effects on flora and fauna in their introduced ranges. We show that *Alliaria petiolata*, a Eurasian invader in North American forests, is an attractive oviposition site for adult *Anthocharis midea* butterflies, but it is lethal to larvae when they try to feed on it. The drought stress of hosts had little effect on feeding and survival and older larvae were no better than young larvae at surviving on this plant. At present, *A. petiolata* serves as an ecological trap for *A. midea* butterflies, which may lead to local declines in populations of this butterfly and drive selection for the altered behavior of adults to avoid this plant or for larvae to better tolerate it.

## 1. Introduction

The introduction of a non-native plant species to a new habitat can have several direct consequences on the fitness of native herbivores. First, the successful recognition and utilization of the plant by a native herbivore could directly benefit the fitness of the herbivore. Second, an herbivore may fail to recognize a potentially suitable host, resulting in neutral direct effects on fitness. Lastly, the novel host may be recognized by the herbivore but prove unsuitable for feeding and growth, resulting in negative direct effects on fitness [1]. This latter response has been labeled an “ecological trap” or an “evolutionary trap” and has been observed in several plant–herbivore systems often involving non-native plants where the fitness impacts are often severe [2]. *Alliaria petiolata*, garlic mustard (Brassicaceae), is a Eurasian biennial herb that is invasive throughout North American forest understories with numerous direct and indirect effects on native flora, fauna, and microbes [3,4,5,6,7]. Co-occurring in habitats with *A. petiolata* exist several native spring ephemeral plant relatives that host herbivores specializing on brassicaceous plants. For example, native brassicaceous species such as *Cardamine concatenata, Cardamine diphylla*, and *Cardamine pratensis* are hosts for specialist pierid butterflies, such as mustard white butterfly, *Pieris oleracea*, West Virginia white butterfly, *Pieris virginiensis*, and falcate orangetip butterfly, *Anthocharis midea*. The increasing presence of *A. petiolata* in the habitat of such butterfly species increases the chance that they will encounter and attempt to use this plant, with potentially negative (or at least uncertain) fitness effects [1,6].

*Alliaria petiolata* has morphological traits that adult herbivores generally find attractive, including a larger size and more abundant floral display than native relatives. Of particular concern in this case is the novel phytochemical profile of *A. petiolata* relative to native North American relatives. *Alliaria petiolata* is the only member of its genus in the world and has no close native relatives in the Brassicaceae in North America. Consequently, *A. petiolata* shares little overlap in its secondary metabolite profile with native relatives in North America [8]. For example, *A. petiolata* produces alliarinoside, a hydroxynitrile glucoside largely unknown from other species, and glucosinolates, predominantly sinigrin (allylglucosinolate) [9,10,11,12]. While native species possess a variety of glucosinolates, sinigrin that is abundant in *A. petiolata* is absent or at very low levels in the native Brassicaceous species that have been examined, while native Brassicaceous species contain some flavonoids that *A. petiolata* lacks [8]. For any specialist herbivore attempting to use *A. petiolata*, this novel profile of secondary metabolites may have consequences for adult oviposition preferences, the feeding and growth of larvae, and potentially adult performance.

In a well-studied example in this system, Davis and Cipollini [1] showed, in both field settings and in laboratory settings controlling for plant size, that adult *P. virginiensis* butterflies preferentially oviposit on *A. petiolata* over their primary host plant, *C. diphylla*. The attempted use of *A. petiolata* as a host plant by the univoltine *P. virginiensis* is a mismatch event for this butterfly as all larvae die on it after attempting to feed [1]. Two key foliar metabolites from *A. petiolata*, sinigrin and alliarinoside, were found to negatively impact larval survival, leaf consumption, and larval mass when painted on leaves of an otherwise acceptable host, with the effects of alliarinoside being more severe [13]. For this species, *A. petiolata* appears to be an important direct threat to the success of this rare and declining butterfly and an “ecological trap” [1,2]. It may also be an important indirect threat through its negative competitive effects on the abundance of native plants that may serve as adult nectar sources or larval host plants [6].

The related species, *Pieris oleracea*, exhibits differential responses to *A. petiolata* as a host based on the historical exposure of the population to this plant. In areas where *A. petiolata* was a recent invader, there was a large range in preference and survivorship on *A. petiolata* with most larvae performing poorly [6]. In areas with long-established *A. petiolata* populations, *P. oleracea* showed better larval performance on *A. petiolata*, although survival was still generally low. This divergent response of *P. oleracea* to *A. petiolata* indicates that native herbivores may be capable of adapting in both larval performance and adult oviposition preference to the invader given sufficient exposure time. The multivoltine life history of *P. oleracea* may permit more rapid adaptation to novel hosts than for an insect with a univoltine life history.

In this study, we explored adult and larval responses of *Anthocharis midea* to *Alliaria petiolata* as a potential host. This butterfly is a related and more common butterfly than the two *Pieris* species that have been studied in this system, but few observations and no data exist on its response to *A. petiolata. Anthocharis midea* is univoltine and generally lays its eggs singly [14]. Like many pierid butterflies, it displays the red egg syndrome [15]. A key host for this butterfly in forest understory habitats is *Cardamine concatenata*. It oviposits on developing flowers and upper stems of this plant, and larvae consume flowers, young leaves and developing siliques prior to moving to other tissues as needed. Casual observations have indicated that *A. midea* will gather nectar and oviposit on the much larger *A. petiolata* in the field, and that its larvae, as seen with *P. virginiensis*, die upon the initial feeding on this novel host, but few observations and no published data exist. Conversely, *A. petiolata* is an important adult and larval host plant for the closely related orangetip butterfly, *Anthocharis cardamines*, in Europe [8]. Here, we compared the oviposition of *A. midea* in the field on its native host, *C. concatenata* to that on *A. petiolata*. The preference–performance hypothesis (also known as the “mother knows best” hypothesis) states that adult females will preferentially select oviposition hosts and sites that maximize the fitness of their offspring [1]. We hypothesized that *A. midea* would preferentially oviposit on its native host species over *A. petiolata* to maximize the fitness of their offspring.

Larval preference and performance on native and non-native host plants were also examined for *A. midea*, as was the effect of environmental stress on the suitability of host plants. We tested larval performance in both choice and no-choice feeding bioassays. Like adult oviposition preferences, larvae will often choose the best host plant on which to feed when given a choice. However, when confined to only one host, effects can range from acceptance and successful feeding and growth and acceptance followed by toxic effects to outright rejection and starvation [10]. We predicted that *A. midea* larvae would choose, feed and survive best on their native host. In addition, environmental stress, like drought, can result in changes in plant chemistry that can influence the interactions between host plants and herbivores that utilize them [16]. For example, the exposure of *Brassica oleracea* to drought conditions led to changes in volatile production and chemical defense levels [17]. When *Mamestra brassicae* (Noctuidae) moths were presented with drought-stressed and well-watered *B. oleracea*, they preferred to lay eggs on drought-stressed individuals [17]. The glucosinolate concentration of drought-stressed *A. petiolata* was substantially lower than that of well-watered *A. petiolata* [18]. When presented with drought-stressed and well-watered plants, the larvae of the specialist herbivore *Pieris brassicae* (Pieridae) preferred to feed on well-watered plants, while *Spodoptera littoralis* (Noctuidae), a generalist herbivore, preferred drought-stressed plants. Despite its preference for well-watered plants, *P. brassicae* developed faster on drought-stressed plants [18]. We predicted that drought stress would increase the preference for and ability of *A. midea* larvae to feed and grow on leaves of *A. petiolata* but that drought stress would make their native host less preferred and of a lower quality.

As larvae mature, gain mass and become more mobile, they may exhibit changes in feeding preference or capability as resources from their host are exhausted. In general, larvae can become more tolerant to defensive compounds produced by host plants as they mature [19], suggesting that some previously unacceptable host tissues or plants may become acceptable to late instar larvae. In *A. cardamines*, which specializes on *A. petiolata* in its native range, adults oviposit and larvae subsequently feed mainly on floral parts or siliques of their host rather than leaves [20], but they will increasingly move around a plant to other tissues as they mature [9]. Once food resources are exhausted from their relatively small host plants, increasingly mobile late instars of *A. midea* must move to other plant parts or neighboring plants to complete development. When native and novel species are in close proximity, there is potential for larvae to move from a suitable native to a novel species on which their success is uncertain. We predicted that later instars of *A. midea* would be more capable of feeding and surviving on *A. petiolata* than earlier instars.

## 2. Materials and Methods

### 2.1. Butterfly Life Cycle

*Anthocharis midea* is a univoltine butterfly distributed throughout the southeastern and southcentral U.S. that overwinters as a chrysalis and emerges in early Spring (typically April in its range in Ohio). Adults forage for nectar from a variety of plants, including their brassicaceous host plants, and live for approximately one week. After mating, they generally lay eggs singly on preferred host plants (but see below) and eggs hatch within one week. Larvae feed for up to 14 days and then pupate near the base of their host plants or on trees [21].

### 2.2. Oviposition Preference in the Field

Adult oviposition preference was examined at Scioto Trail State Forest (39.209766717151716, −82.95566822332384) near Chillicothe, Ohio, on 14 April and 21 April 2016 through paired-plant comparisons. This forest consists largely of oak–hickory forests with *C. concatenata* growing abundantly throughout the understory. *Alliaria petiolata* is less abundant and is present primarily in scattered patches adjacent to forest roads and trails. Observation plots were selected by driving on the forest roads, identifying ~10-m^2^ patches where at least twenty flowering individuals of each host grew in an intermingled fashion (*C. concatenata* was always more abundant). Plots were chosen haphazardly throughout the forest, and no two plots were within 100 m of each other. All *A. petiolata* detected (typically ~25) and an equal number of *C. concatenata* in the plots were visually searched for *A. midea* eggs. Eggs are usually placed singly on plants, but there can be multiple eggs placed on plants in some instances. Ten plots were examined on April 14 and 13 plots were examined on 21 April. On each date, the number of plants of each host in the plot with eggs and the number of each host with multiple eggs were recorded. Different plots were observed on each date but in similar areas. While the smaller, perennial *C. concatenata* flowers earlier than the larger, biennial *A. petiolata*, the major difference in host plant status between the two dates was that *A. petiolata* had floral buds but was not fully flowering on 14 April, while it was flowering more prominently on 21 April. Conversely, *C. concatenata* was in full flower on 14 April but was past its flowering peak by 21 April. These differences were not precisely quantified. An oviposition preference index (OPI) at the plot level was calculated by subtracting the number of *A. petiolata* plants with eggs from the number of *C. concatenata* plants with eggs divided by the total number of plants with eggs in the plot.

A positive value indicates a preference for the native host, while a negative value indicates a preference for the novel host. The OPI was analyzed with a one-sample *t*-test; in a system without preference where the OPI was zero, the *t*-test established if the observed values were significantly different from zero. The independent and interactive effects of the survey date and host species on egg number and the number of plants in each plot receiving multiple eggs were analyzed using ANOVA. All statistical analyses for this and subsequent analyses were completed using RStudio (Version 1.1).

### 2.3. Egg and Plant Collection

Eggs of *A. midea* for larval bioassays were collected from *A. petiolata* on April 21 as the oviposition surveys were conducted by removing them with as little host plant tissue as possible. All eggs were moved immediately to leaves of *C. concatenata* in plastic lidded containers and kept in a cooler until their return to the laboratory. Larvae generally hatched from these eggs within one day and were reared in an incubator on *C. concatenata* leaves for five days prior to bioassays.

*Cardamine concatenata* and *A. petiolata* plants used to provide material for larval bioassays were collected from the same general location in the Wright State University Woods (39.78356626482252, −84.05868811970556) in March 2016. Plants were transplanted into 6-inch pots in ProMix BX potting soil in the greenhouse at Wright State University and grown as described below. Only second year *A. petiolata* (n = 92) and flowering *C. concatenata* plants (n = 192) were collected as these plant stages are primarily targeted by adult butterflies. While growing conditions prior to collection may have varied somewhat, replicate plants of each species were grown for one month under one of three watering conditions, either well-watered, exposed to moderate water limitation, or exposed to severe water limitation. We used a practical method to impose water stress that involved the visualization of wilting as a sign of water limitation [22]. Well-watered plants were provided with water such that no evidence of wilting was ever visible. Moderate water limitation was achieved by watering plants only when 50% of the plants in this treatment showed signs of wilting. Severe water limitation was achieved by watering plants only when 75% of the plants in this treatment showed signs of wilting. Plants were watered just prior to using them in bioassays, and leaves used in bioassays were chosen haphazardly from the replicate plants available in each treatment. There was a chance that some plants from which leaves were used in bioassays had not been particularly water stressed, even in water-limited treatments.

### 2.4. Larval Performance in No-Choice Bioassays

To test the effects of host species and drought stress on *A. midea* larval development, larvae were enclosed singly in containers with leaves of one host species and drought stress treatment. Bioassays were set up in Petri dishes, each containing a half a piece of Kim-Wipe folded into a small square and saturated with distilled water. Leaves were offered to single larvae in each dish in as close to the same mass in each container as possible. To do this, leaves of similar sizes were chosen for bioassays or if flowering material was used, the same volume of buds was used. The amount of material provided to the larva in performance assays was also adjusted to the instar of the larva to avoid any food limitation and dishes were cleaned as needed. After setting up bioassays, dishes were lidded, sealed with parafilm, and placed on an open bench in a laboratory maintained at ~22 °C with windows allowing in natural light and with fluorescent lights on between the hours of ~0900 and 1700. Larvae were very small but of similar size at the onset of the bioassay and not weighed as they were difficult to handle. Survival of larvae was monitored through time and larvae were weighed at the conclusion of the 14-day experiment or when they died. Feeding was visually observed but not quantified. The different treatments provided to *A. midea* larvae included tissues of well-watered *C. concatenata* (n = 8) and *A. petiolata* (n = 7), moderately water limited *C. concatenata* (n = 8) and *A. petiolata* (n = 9), and severely water-limited *C. concatenata* (n = 8) and *A. petiolata* (n = 9). Survival of larvae was analyzed using the package *survival* in R studio and displayed using Kaplan–Meier curves. ANOVA was used to analyze how larval mass was influenced by host species and water stress level of hosts.

### 2.5. Larval Preference in Choice Bioassays

Choice tests were conducted to identify the feeding preferences of *A. midea* larva for leaves from different host species or plants exposed to different water availability. To test the relative preference for native and non-native hosts, single larvae enclosed in Petri dishes as above were presented with the foliage of *C. concatenata* and *A. petiolata* at the same time. The foliage of a similar mass was placed on either side of the dish and then a larva was introduced between the foliage. To test the preference for foliage from plants exposed to different water availabilities, larvae were enclosed with foliage from severely water limited and normally watered plants, separately for each species. Due to egg limitation, choice bioassays included 4–5 replicates of each treatment and species combination. Preference for each host species or plant water status was recorded by the presence of the larva on the tissues and a visual assessment of the degree of feeding from them over a period of 6 days. Larval choices were universally established within one day and persisted at generally the same level through the length of the bioassay; therefore, we present and discuss only the results after one day when all larvae had made their feeding choices and were still alive. These data were not statistically examined.

### 2.6. Host Transfer

To observe whether older, more mobile larvae were capable of feeding successfully on *A. petiolata*, ten *A. midea* larvae were first allowed to feed on *C. concatenata* tissues in Petri dishes as described above for 10 days and then transferred to leaves of *A. petiolata*. Larval masses were recorded periodically and days to death were recorded until all larvae expired.

## 3. Results

### 3.1. Oviposition Preferences in the Field

**Figure 1:**
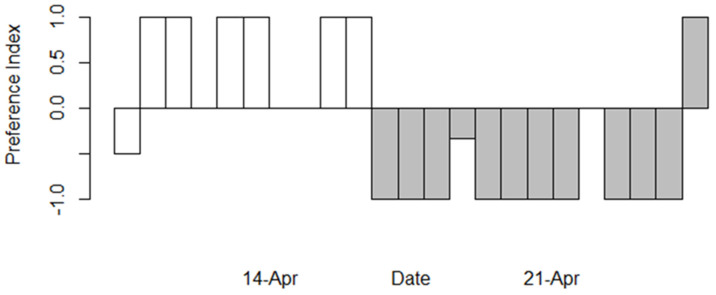
Oviposition preference indices of *Anthocharis midea* for native and non-native hosts at Scioto Trail State Forest on two dates in April 2016. A positive index indicates a preference for the native host, *Cardamine concatenata*, while a negative index indicates a preference for the non-native host, *Alliaria petiolata*. Each bar represents the preference index for each plot sampled. Bars appearing to be absent = 0. N = 10 on 14 April (open bars) and N = 13 on 21 April (gray bars).

**Figure 2:**
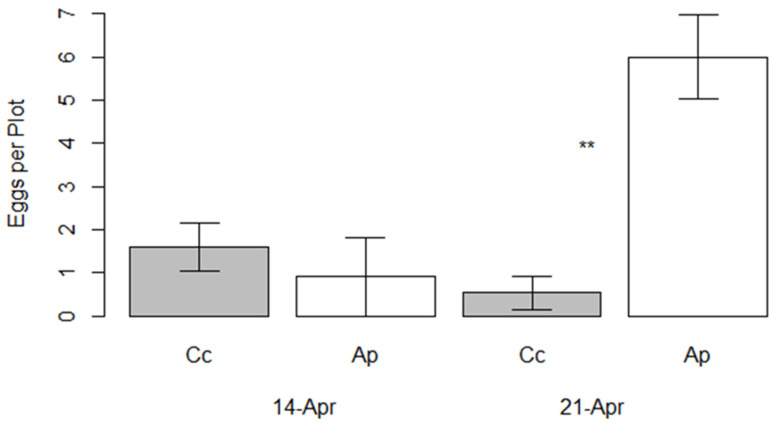
Mean number of eggs (±1 SE) of *Anthocharis midea* per plot placed on *Cardamine concatenata* (Cc) and *Alliaria petiolata* (Ap) on two dates in 2016 at Scioto Trail State Forest. Asterisks indicate a significant difference between hosts at α = 0.05.

Oviposition preference of *Anthocharis midea* was affected by host species and observation date. Earlier in the season, *A. midea* displayed a preference for *C. concatenata* (t = 2.906, *p* = 0.017), while a week later in the season, the preference shifted strongly toward *A. petiolata* (t = −4.27, *p* = 0.001, Figure 1). Across the season, *A. petiolata* received more eggs than *C. concatenata* (Species: F = 0.028, *p* = 0.003, Figure 2) and more eggs were laid later in the season than earlier (Date: F = 5.191, *p* = 0.0278). Slightly more eggs were laid on *C. concatenata* earlier in the season, but substantially more eggs were laid on *A. petiolata* later in the season (interaction: F = 10.028, *p* = 0.003).

While the presence of multiple eggs laid on a single plant was observed occasionally by us on native hosts in this forest, multiple egg events were observed on only *A. petiolata* in the paired-plot comparisons. When multiple egg events were seen on native individuals, the eggs were few in number and spaced widely apart. Multiple egg events seen on *A. petiolata* individuals in our survey were observed both at distal locations on the plant but more often clustered on the same floral structure or young leaf. The occurrence of multiple oviposition events was significantly influenced by host species (F = 4.442, *p* = 0.041) but not by date (F = 2.555, *p* = 0.117).

### 3.2. Larval Performance in No-Choice Bioassays

**Figure 3:**
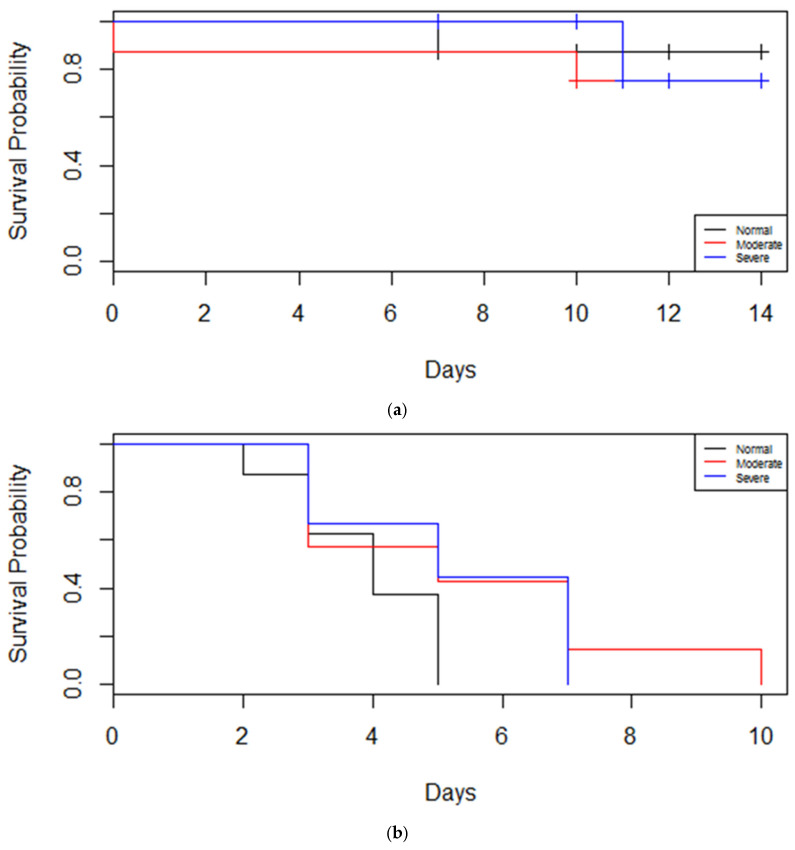
Survival probabilities of *Anthocharis midea* larvae through time on tissues of hosts given one of three levels of drought stress. (**a**) Survival of *A. midea* on the *Cardamine concatenata* under three levels of drought stress, none (black), moderate (red) and severe (blue). (**b**) Survival of *A. midea* on the *Alliaria petiolata* under three levels of drought stress, none (black), moderate (red) and severe (blue).

Larvae fed extensively on *C. concatenata*, as expected, and about 80% of them survived to the end of the 14-day bioassay regardless of the drought status of their hosts (Figure 3a). While not quantified precisely, larvae fed continuously on *C. concatenata* throughout the time they were alive. Feeding was minimal and survival was significantly lower on *A. petiolata* overall and half of all larvae were dead by five days (F = 65.9, *p* < 0.001, Figure 3b). Feeding on *A. petiolata*, when it occurred, typically consisted of a small feeding attempt (~1 mm diam hole) by a larva followed by a cessation of feeding. No larvae on *A. petiolata* survived to the end of the bioassay. Drought stress extended the duration of the survival of the larvae on *A. petiolata* to some degree, but mortality was still complete across all treatments and survival was not significantly affected by drought status across species (F = 0.414, *p* = 0.663, Figure 3b).

**Figure 4:**
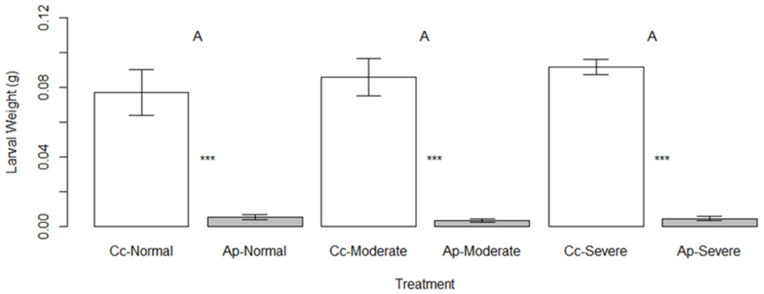
Mean larval mass (±1 SE) of *Anthocharis midea* after feeding on tissues of *Cardamine concatenata* (Cc, open bars) and *Alliaria petiolata* (Ap, gray bars) at three different levels of drought stress (normal, moderate, and severe). Asterisks indicate differences among hosts at each level of drought stress. No significant differences existed among drought levels.

As with the pattern seen in survival, larval masses were much greater on the native host than on the novel host (F = 236.829, *p* < 0.001) but drought stress had no significant impact on larval mass across species (F = 0.816, *p* = 0.449, Figure 4). Larvae increased in mass approximately ten-fold on *C. concatenata* during the duration of the bioassay but failed to grow and died on *A. petiolata*.

### 3.3. Larval Preference Bioassays

Larval choice experiments were not well replicated, but the patterns were very clear. When offered tissues of both hosts simultaneously, *Anthocharis midea* larvae showed a strong preference for *C. concatenata* over *A. petiolata* (Table 1). With one exception, larvae moved quickly to *C. concatenata* and stayed there feeding throughout the duration of the bioassay. One larva chose *A. petiolata* but did not feed or survive for long on it. Larvae offered a choice of severely drought-stressed or normally watered *C. concatenata* showed a strong preference for normally watered tissues (Table 1). In contrast, when offered a choice of severely drought-stressed and normally watered *A. petiolata*, larvae displayed no feeding preference. All feeding from *A. petiolata*, when it occurred, was at a much smaller degree than on the native *C. concatenata*, as noted above, and the larvae that fed on *A. petiolata* generally died quickly.

### 3.4. Host Transfer

For the larvae of *Anthocharis midea* transferred from their native *C. concatenata* to the non-native *A. petiolata* 10 days into development, feeding was minimal or absent on *A. petiolata*, and movement was generally arrested. All but one of these larvae died within 12 days. The one that survived to pupation fed minimally at the start of the bioassay, then commenced pupation shortly after transfer.

## 4. Discussion

The introduction of non-native species to novel habitats can have numerous consequences for native ecosystems, including the disruption of co-evolved interactions between specialist herbivores and their host plants [1]. We explored the effect of *Alliaria petiolata* on the oviposition preference and larval performance of *Anthocharis midea*, a butterfly that uses native spring ephemeral mustards as hosts in North America. We additionally explored the effect of larval age and plant stress on larval feeding preferences and performance.

While *A. midea* showed a significant oviposition preference for its native host *C. concatenata* earlier in the flight season, this preference shifted strongly to *A. petiolata* after one week. Butterflies undergo several behaviors to identify a suitable host, including searching, orientation, encounter, landing, surface evaluation and then acceptance [23]. During the searching phase, the cues indicating a suitable host are mostly visual, extending from the shape and color of the plant to the apparency of the host [20]. There were distinct differences in the phenological stage of the host plants on the dates surveyed in this study, which are likely important given that *A. midea* prefers to lay eggs on floral structures and upper leaves of the plant. On the first survey date, the native host was in full bloom and received the majority of oviposition events, while the novel host was not yet blooming and received fewer oviposition events. On the second survey date, *A. petiolata* was in bloom and received the majority of oviposition events, while the majority of the *C. concatenata* encountered were past peak flowering and received few oviposition events. This same seasonal increase in the selection of *A. petiolata* as an oviposition site has been observed for *Pieris virginiensis* butterflies on *A. petiolata* in West Virginia [24]. Floral structures of the two plants we studied are similar and both species produce glucosinolates. These floral structures could serve as a visual cue for a suitable host, and the similar chemosensory profiles of the two hosts could lead to mismatch oviposition events. However, flowering stalks of *A. petiolata* can also reach 1 m in height [25], which is far taller than the 20 cm that the native *C. concatenata* averages, and there are many more flowers in the branched inflorescences of *A. petiolata*. In the European orangetip butterfly, *Anthocharis cardamines*, the number of flowers on an inflorescence strongly influences oviposition rates on *Cardamine pratensis*, a common host plant of this butterfly [26]. The visual and biochemical apparency of large flowering *A. petiolata* plants may serve as a supernormal stimulus for *A. midea*, resulting in the preferential selection of this host when it is flowering. This may be true both for adults foraging for nectar and for those seeking oviposition sites.

As observed in studies on related *Pieris* species, larvae of *A. midea* were unable to survive on tissues of *A. petiolata* and generally fed very little on it. While we did not explore the mechanism of the lethal effects of *A. petiolata*, it is likely due to the same compounds that affect *Pieris* species, namely high levels of some novel glucosinolates not found in their host plants, like sinigrin, and the presence of novel compounds, like alliarinoside, that are lethal to *Pieris virginiensis* larvae [8,13]. Having an oviposition preference for *A. petiolata* but with lethal effects on larvae supports the assertion that *A. petiolata* currently serves as an ecological trap for *A. midea* and may result in local declines in the abundance of this butterfly in areas where *A. petiolata* is abundant. In the forest that we studied, *A. midea* is still commonly observed, but *A. petiolata* is not very abundant relative to *C. concatenata*. The influence of *A. petiolata* on this butterfly could be more profound in areas where *A. petiolata* is more abundant.

Mismatch oviposition events on lethal hosts may have other repercussions for populations of *A. midea*, including shifts in butterfly phenology and host plant use. Host plant associations play a profound role in the ecology and evolution of butterflies; shifts in the usage of chemically distinct plant groups alters population structure and drives the evolution of different butterfly species [27]. In a related species, individuals from populations of *Pieris olereacea* exposed to *A. petiolata* for longer periods of time show a preference for *A. petiolata* as a host and a slight increase in the performance of the offspring relative to those more recently exposed [6]. This response supports the idea that adaptation in both host preference and larval performance may be possible with regard to *A. petiolata* use by some native butterflies, especially those that are multivoltine. If *A. midea* continues to differentially select hosts at different times during the mating season, at least two outcomes could occur. In one case, early emerging genotypes that are more likely to oviposit on an acceptable host may be selected for as the offspring of later emerging and ovipositing adults that choose *A. petiolata* will perish. Population shifts toward genotypes with an earlier spring phenology to avoid *A. petiolata* would be constrained, however, by risks due to the exposure of populations to inclement weather and further constrained if the phenology of *A. petiolata* also shifts earlier in response to warmer springs. Second, if the temporal separation in host selection continues, the population could diverge into two distinct races, one that specializes on early-flowering mustards, like *C. concatenata*, and one that specializes on later-flowering mustards, like *A. petiolata*. This would be possible only if the butterflies were to experience some adaptation that allowed them to utilize *A. petiolata* as a larval host. Thus far, there is no evidence that *A. midea* larvae can survive on *A. petiolata*, although the European orangetip butterfly, *A. cardamines*, readily uses it as an adult and larval host [9,26].

*Alliaria petiolata* enhanced the frequency of multiple oviposition events for *A. midea*, which are not typically seen for this species [14]. The red egg syndrome exhibited by *A. midea*, as for many other pierid species, is a mechanism to signal to other searching adults that a host is occupied [15]. Only a few instances of multiple oviposition events (also including lower numbers of eggs) were observed on *C. concatenata* while many multiple events, with the eggs laid in higher numbers, were observed on *A. petiolata*. If *A. petiolata* serves as a supernormal visual or chemical stimulus, this could explain the deviation from the typical behavior and indicates that many offspring can be negatively affected at once. The more frequent occurrence of multiple oviposition events at the later survey date could also be explained as a function of time during the reproductive season. Adult flight periods of individual butterflies are only about one week long so decisions need to be made quickly by gravid adults. Earlier in the season, females may be ‘choosier’ as selecting the best host would be of the greatest benefit to larval survival. Towards the end of the reproductive season, this ‘choosy’ behavior may not be as important as the benefit of ovipositing as many eggs as possible. However, this effort backfires when the selected host is lethal to larvae.

Specialist herbivores typically feed from a narrow range of plants [28]. Specialists are well equipped to metabolize the secondary defenses of a specific group of plants, but they are unable to utilize a wide array of plants. When a host is stressed, however, plants may be unable to allocate as many resources to developing secondary defenses, lowering defensive chemical concentrations [29]. While drought is an uncommon occurrence in the spring in temperate forests, it can happen, and plants can also grow in soils that vary naturally in moisture availability. Regardless, stressors like drought can be used as a tool to assess whether stress-induced changes in plant quality are sufficient to alter plant–insect interactions [16,17,18]. Larvae were generally able to freely feed, survive, and reach pupation on their native host in this study, regardless of the drought status of the plants. When given a choice, however, *A. midea* larvae strongly preferred tissues from well-watered native hosts over those from droughted plants, suggesting that there was some impact on tissue quality or attractiveness caused by the drought and sensed by larvae. Conversely, drought stress tended to extend the duration of the feeding and survival of *A. midea* larvae on *A. petiolata*, although all larvae still died, but larvae did not discriminate between leaves of healthy and droughted plants when given a choice. Shifts in secondary metabolites due to drought stress in *A. petiolata*, if they occurred, were not great enough to allow for the novel *A. petiolata* to be a suitable host for larvae in this study. However, drought or other stressful conditions could facilitate the increased survival of larvae on this novel host in the field to the point where motile larvae could periodically use it, as long as other suitable hosts for young larvae are nearby. In some insects, older larvae are able to better handle chemical defenses of their hosts than younger larvae [18], which is useful as older larvae may be forced to seek other hosts if resources from their original host are exhausted. We found no evidence, however, that later instars of *A. midea* had an increased capacity to utilize *A. petiolata*. The one older larva that survived on *A. petiolata* in our bioassay did so only by foregoing feeding and pupating at an earlier age than is typical for this species.

## 5. Conclusions

As for other pierid butterflies studied so far, we show here that *Anthocharis midea* adults will oviposit on *Alliaria petiolata*, at times preferentially so, but larvae cannot survive on this plant and will avoid feeding on it when given a choice. Drought stress slightly alters these relationships but had no significant effect on survival and growth. The mechanism of larval rejection and failure is currently unknown but likely involves compounds lethal to other pierid butterflies [13]. At present, *A. petiolata* serves as an ecological trap for *A. midea*, as has been shown for other pierid species [1,6], and could negatively impact populations of this butterfly where *A. petiolata* is abundant, but the long-term ecological and evolutionary impacts of *A. petiolata* on *A. midea* require further study. *Alliaria petiolata* has long been a target for control efforts in North America, primarily because of its purported competitive impacts on other plants [25], but the results shown here and in other studies [1,6] indicate that its negative impacts on specialist herbivores alone make it worthy of management. The results of this study highlight the threats to biodiversity of the introduction of novel species to native habitats, which are likely more common in co-evolved plant–herbivore systems than we realize.

## Figures and Tables

**Table 1:** Feeding preferences of individual *Anthocharis midea* larvae. Asterisks indicate a preference toward the lefthand choice, a hyphen indicates a preference toward the righthand choice, and a 0 indicates no preference. The number of times the symbol is repeated indicates the intensity of the response.

Pair	Choice
Native vs. Invasive	***
Native vs. Invasive	***
Native vs. Invasive	***
Native vs. Invasive	***
Native vs. Invasive	-
Normal vs. Droughted *C. concatenata*	***
Normal vs. Droughted *C. concatenata*	***
Normal vs. Droughted *C. concatenata*	***
Normal vs. Droughted *C. concatenata*	***
Normal vs. Droughted *A. petiolata*	0
Normal vs. Droughted *A. petiolata*	-
Normal vs. Droughted *A. petiolata*	0
Normal vs. Droughted *A. petiolata*	-

## Data Availability

The raw data supporting the conclusions of this article will be made available by the authors on request.
